# ArtsRx as an arts prescribing model: leveraging CRM data to understand participation and engagement

**DOI:** 10.3389/fpubh.2026.1800466

**Published:** 2026-06-10

**Authors:** Kristen D. Krause, Ricardo Kairios, Taylor Masamitsu, Kendall Lyra, Aly Maier Lokuta

**Affiliations:** 1 Center for Health, Identity, Behavior, and Prevention Studies, School of Public Health (CHIBPS), Rutgers University, Newark, NJ, United States; 2 Department of Urban–Global Health, School of Public Health, Rutgers University, Newark, NJ, United States; 3 Department of Family and Community Health Sciences, Rutgers Cooperative Extension, Rutgers, The State University of New Jersey, New Brunswick, NJ, United States; 4 New Jersey Performing Arts Center (NJPAC), Newark, NJ, United States; 5 The Colton Institute of Arts Training and Research at NJPAC, Newark, NJ, United States

**Keywords:** arts and health, arts prescribing, community-based intervention, customer relationship management, digital health, engagement, health equity, social prescribing

## Abstract

**Introduction:**

Arts prescribing programs, through which health and social care providers refer patients to arts-based activities to support health and wellbeing, are emerging in the United States but remain heterogeneous in design and implementation. Moreover, many community-based models lack consistent, technology-enabled engagement tracking systems for tracking attendance, engagement, and longitudinal participation, limiting evaluation and quality improvement. To address this gap, ArtsRx, a program developed and managed by an anchor cultural institution in New Jersey, leverages a customer relationship management (CRM) platform to capture real-time registration, attendance, and engagement data. This study presents a 2-year analysis of ArtsRx participation patterns and modifiers of engagement.

**Methods:**

This retrospective observational study was done via quantitative analysis of de-identified records for 498 individuals referred to ArtsRx between 2023 and 2025. Data extracted from the program's CRM were used to categorize engagement (no, low, medium, high). Descriptive statistics were calculated for engagement rates and examined by demographic characteristics (age, race/ethnicity, gender) and referral source.

**Results:**

Among 498 participants, 21.9% completed the program, 45.7% withdrew, 19.3% remained enrolled, and 13.1% submitted an intake form without fully enrolling. Engagement analyses focused on 336 participants who completed or withdrew. Completers attended a mean of 78.2% of scheduled activities, compared with 8.7% among withdrawn participants. Engagement level was associated with program year and referral source but not with race/ethnicity or gender; referral patterns varied by program year and participant demographics.

**Conclusions:**

This analysis demonstrates the feasibility of a CRM-based platform for monitoring engagement in U.S. arts prescribing programs. Participation patterns indicate consistent engagement across demographic groups, with variations linked to referral pathways and program year. These findings support the use of technology-enabled infrastructure for strengthening and scaling arts prescribing models within health and community care systems.

## Introduction

Social prescribing has emerged as an innovative, person-centered approach to addressing the social determinants of health by connecting individuals with non-clinical resources to improve wellbeing while reducing strain on traditional healthcare systems (1–3). In these ways, social prescribers recontextualize some traditional medical interventions by emphasizing that many health concerns, such as loneliness, stress, and mild to moderate mental health conditions, are rooted in social determinants of health, including social isolation, housing instability, and lack of community engagement (4–6). In practice, social prescribing typically involves healthcare or community professionals referring individuals to a connector or “link worker,” who co-produces a personalized plan and connects the person with local activities and support services (1). These plans may include arts and cultural programs, exercise groups, volunteering, gardening, and/or financial counseling (1). Across plans and participants, the goal in social prescribing is to empower individuals to take control of their health, build social connections, and improve quality of life through meaningful engagement (7).

Social prescribing is most established in the United Kingdom. In studying these programs' respective outcomes, scholars suggest that these programs can reduce psychological distress, alleviate loneliness, enhance social cohesion, and decrease unnecessary healthcare utilization (7, 8). These findings, largely drawn from long-term, primary care–embedded initiatives, are useful for practitioners and policymakers developing social prescribing in other industrialized contexts, including the United States (U.S.) (7). However, the evidence base remains mixed and limited by methodological challenges, such as heterogeneous program models and over-reliance on self-reported outcomes (7). In the U.S., social prescribing is still emerging, and rigorous evaluations are needed to clarify which populations social prescribing practitioners are best able to support and under what circumstances (9). Importantly, social prescribing advocates and practitioners view these programs as a strategy to advance health equity by addressing social determinants that drive disparities in health outcomes. Yet, barriers such as transportation, cultural relevance, and accessibility for marginalized groups can undermine its potential impact, highlighting the need for thoughtful implementation and ongoing monitoring (9).

Arts prescribing is a subset of social prescribing, in which prescribers intentionally connect people with arts, culture, and creative expression as a means to improve health and wellbeing (10).The first recognized arts prescribing program in the U.S. is CultureRx, launched as a pilot in Massachusetts in 2020 (11). Since then, and with increasing frequency, arts prescribing programs are developing across the U.S., focused on a variety of populations and at various sizes and scopes (9, 12). The existing evidence from these programs suggests positive impacts on health and social connectedness, though there is substantial opportunity for further study to establish standardized engagement metrics, strengthen data infrastructure, and conduct rigorous evaluations in U.S.-based, community-centered arts prescribing models (10). Scholars evaluating social prescribing initiatives report improvements in loneliness and wellbeing for a substantial proportion of participants. For example, one large mixed-methods evaluation found that approximately 70% of service-users reported feeling less lonely after participating in a link worker social prescribing program, with mean UCLA loneliness scores improving by about 2 points (13). Other reviews of social prescribing and arts-on-prescription programs describe statistically significant gains in mental wellbeing and reductions in anxiety and depression, alongside selective evidence of reduced primary care use and emergency department attendance in some settings (8, 10).

Indeed, despite growing recognition of their benefits, evaluating arts prescribing programs has revealed similar challenges with broader social prescribing initiatives—namely, through a lack standardized methods for monitoring engagement and evaluating outcomes (9, 10, 12). Few community-based, community-centered U.S. arts prescribing initiatives have systematically assessed participation patterns using technology-enabled engagement tracking systems (9, 12). This gap limits opportunities for implementation science, policy development, and scalability. Digital health tools, including customer relationship management (CRM) platforms, present a promising solution for real-time data capture and longitudinal evaluation, yet their application in arts prescribing remains underexplored.

In 2022, NJPAC Arts & Wellbeing began to develop plans for an arts prescribing program to serve Newark and Essex County, New Jersey, U.S. in partnership with insurance carrier Horizon Blue Cross Blue Shield of New Jersey (Horizon). This marked the first cultural organization-health insurer arts prescribing partnership in the U.S. Over 10 months of planning, NJPAC and Horizon worked together to create a referral pathway, participant pre- and post-surveys, referrer training materials, a network of engaged arts partners, and—critically—a custom Program Management Module within their CRM platform Salesforce (specifically: Salesforce Sales cloud with Non Profit Success Pack) to track the journey of every participant engaged in the program. ArtsRx launched in September 2023 with Horizon as its inaugural referral partner, receiving referrals from community health workers and case managers already working with Horizon members experiencing complex needs around their social determinants of health. By embedding arts engagement within a social determinants of health framework, ArtsRx reinforces that access to arts and culture is a social driver of health (14), and demonstrates how partnerships between healthcare, community organizations, and cultural institutions can foster holistic wellbeing and behavioral health equity (11).

### ArtsRx referral pathway: description and process

ArtsRx now receives participant referrals from six sources: horizon, two federally qualified health centers (FQHCs) through partnerships with RWJBarnabas Health; the City of Newark Department of Health; a community group for formerly incarcerated individuals and their families (Returning Citizens Support Group); Rutgers University–Newark students; and NJPAC employees. Referrers across these organizations include physicians, community health workers, case managers, nurses, faculty, staff, student-peer support specialists, chaplains, and community liaisons. Unique for the NJPAC employees is a self-referral option. The multiple referral pathways are designed to maximize accessibility and engagement across Newark, NJ and Essex County. NJPAC staff hold trainings for referrers from each partner organization and advise that while everyone can benefit from increased engagement in the arts, the program is especially beneficial for those experiencing social isolation, loneliness, caregiving stress, chronic pain or illness, or those with clinically well-managed mental or behavioral health needs (7, 10). The trainings are held via Zoom and include information on why arts participation is beneficial for health and wellbeing, details of the referral process, and opportunities for practicing making a referral using suggested scripts. The Zoom training recordings are shared with the referrers alongside an FAQ packet and any slides used during the training, and physical business cards with ArtsRx enrollment instructions are delivered which the referrers can give to those receiving referrals.

Information about the ArtsRx program is shared by the trained referrers to potential participants, and participants can sign up for the program by completing an intake form, accessed via phone call, text, email, or QR code. The process includes both “warm handoffs,” where referrers assisted participants in completing the intake form and “cool handoffs,” where participants receive a link via email, text message, or business card and complete the form independently. Participants are assured that all activities offered through ArtsRx are provided at no financial cost to participants, with registration and ticket fees fully covered through NJPAC institutional and philanthropic support.

NJPAC manages the ArtsRx program, serving as the hub for arts referrals, maintaining a network of over 10 local arts organizations so participants can choose from a range of activities, including museum admission, ceramics classes, poetry workshops, or performance tickets. Of note is that participating arts organizations do not have to create any new programming. ArtsRx connects participants with the organizations' existing roster of programs and activities via an ArtsRx connector who works at NJPAC (i.e., link worker). Participants have up to 6 months from enrollment to engage in up to six ArtsRx activities, with an opportunity at the 6-month mark to reassess their goals, complete the intake survey again, and re-enroll. During this period, participants have full autonomy to choose both the number of activities they wish to attend and the specific activities they wanted to engage in. For instance, some select three activities within the 6-month span, while others participate in up to six. This engagement cycle is individualized for each participant and allows longitudinal tracking of participation and re-engagement patterns. After 6 months of enrollment, or after six activities have been completed (whichever occurs first), the participant is sent a post-program survey and marked as completed. If at any time during enrollment in ArtsRx a participant becomes unresponsive to the ArtsRx Connector's communications for over 2 months, the participant is withdrawn from the program.

At every stage in the program and participant management, information is tracked via NJPAC's custom CRM program module, Salesforce (San Francisco, CA). ArtsRx staff inputs records of communication success or failure, type of communication (email, text, phone call), creates records for each arts activity registered for, how many guests were registered and whether the participant (and guests, when applicable) attended. The arts activities are organized by arts organization, tagged by modality (crafts, music, dance/movement, literary arts, performance/theater, and visual arts), and receptive or active engagement. Program costs are tracked by individual registration, total overall cost to-date, total cost for the current fiscal year, and total to-date payments to each arts organization. Attendance rates are tracked by all participants, all participants and guests, by referral source and by arts organization. Attendance rate is defined as how many arts activities the participant attended out of how many arts activities they were registered for. Engagement rates for participants are also tracked. Engagement rate is defined as how many arts activities a participant attended out of the maximum possible six arts activities available during their enrollment. Thus, attendance rate captures participation among scheduled activities, whereas engagement rate captures use of the full six-activity allotment.

While the Salesforce system provides robust data management and visualizations via dashboards, formal quantitative data analysis with regressions was required to investigate patterns of engagement. Leveraging the NJPAC-Rutgers University Arts in Health Research Lab partnership, an application was submitted, and a grant was received from the Rutgers–New Brunswick OVPR Behavioral Health and Equity Pilot Seed Funding, which aims to explore innovative intersections between behavioral health and wellbeing.

This manuscript presents the first quantitative analysis of ArtsRx program data, examining 2 years of participation records to (1) describe patterns of attendance, engagement, and attrition; (2) assess how engagement varies by program year, referral source, and participant demographics; and (3) consider implications for using CRM-enabled engagement tracking to inform implementation and equity-focused scale-up of arts prescribing models. The study was guided by a pragmatist orientation, aiming to generate findings that directly inform program refinement and future research on engagement in arts-based social prescribing.

## Methods and materials

### Study design and participants

This retrospective observational study used de-identified administrative and survey data from the NJPAC ArtsRx arts prescribing program. The dataset comprised 498 individuals referred to ArtsRx between September 2023 and March 2025. Data were collected as part of routine operations and subsequently shared with Rutgers University investigators for secondary analysis. NJPAC program administrators did not participate in data analysis, preserving analytic independence. Data sources included participant intake and post-program surveys administered via Formstack (now Intellistack; Denver, CO) in Year 1 and Qualtrics (Provo, UT) in Year 2, as well as attendance, enrollment, and engagement data captured in the aforementioned custom-built Salesforce CRM module. Attendance and engagement were recorded in real time in Salesforce through activity follow up surveys, and occasionally by NJPAC staff and partner organizations when activity follow up survey data was unavailable.

### Measures

#### Program participation and engagement

Program participation status was categorized at the time of analysis as completed, withdrawn, enrolled, intake form submitted but not enrolled, or missing. Engagement analyses focused on participants who either completed or withdrew (*n* = 336), given their sufficient follow-up to evaluate attendance and engagement outcomes. Engagement was defined using two CRM-derived metrics: (1) attendance rate, the proportion of enrolled events attended, and (2) engagement rate, the proportion of allotted activities used during enrollment. For descriptive analyses (Table 1), these measures were summarized with means and standard deviations. For bivariate analyses (Table 2), participants were grouped into four mutually exclusive engagement levels based on the number of ArtsRx events attended: no engagement (0 events), low (1, 2), medium (3, 4), and high (≥5 events). These thresholds reflect NJPAC's internal criteria for distinguishing minimal, moderate, and sustained participation, which correspond to approximately one-third, two-thirds, and near-full use of the six available activities.

**Table 1:** Program completion, engagement, and demographic characteristics of ArtsRx participants.

ArtsRx program metrics	*n* (%)	*n* (%)	Total program participants
Program completion status (*n* = 498)			
Completed			109 (21.9)
Withdrawn			227 (45.7)
Intake form received			65 (13.1)
Enrolled			96 (19.3)
Missing			1 (0.2)
Reason for program withdrawal (*n* = 227)	Completed participants	Withdrawn participants	Completed and withdrawn participants
Staff withdrew participant due to unresponsiveness or inactivity	—	160 (70.5)	
The participant asked to be withdrawn from the program	—	18 (7.9)	
Missing	—	*49 (21.6)*	
Year of program (*n* = 336)			
Year 1	48 (44.0)	78 (34.3)	126 (37.5)
Year 2	61 (56.0)	149 (65.6)	210 (62.5)
Referral source (*n* = 336)			
Horizon blue cross blue shield of New Jersey	26 (23.9)	49 (21.6)	75 (22.3)
Rutgers University-Newark	47 (43.1)	118 (52.0)	165 (49.1)
RWJBarnabas health	2 (1.8)	6 (2.6)	8 (2.4)
Returning citizens support group	22 (20.2)	32 (14.1)	22 (20.2)
I am an NJPAC employee	7 (6.4)	5 (2.2)	12 (3.6)
City of Newark department of health	5 (4.6)	17 (7.5)	22 (6.5)
Engagement level (*n* = 336)			
No engagement (0 events attended)	8 (7.3)	205 (90.3)	213 (63.4)
Low engagement (1–2 events attended)	46 (42.2)	17 (7.5)	63 (18.8)
Medium engagement (3–4 events attended)	25 (22.9)	5 (2.2)	30 (8.9)
High engagement (5+ events attended)	30 (27.5)	-	30 (8.9)
Attendance rate (attended over enrolled), M (SD)	78.17 (30.11)	8.74 (27.25)	31.26 (43.05)
Engagement rate (attended over allotted), M (SD)	49.08 (33.93)	2.94 (10.10)	17.91 (30.13)
Participant demographics			
Race/ethnicity (*n* = 336)			
Arab, Middle Eastern, or North African	2 (1.8)	1 (0.4)	3 (0.9)
Asian	12 (11.0)	22 (9.7)	34 (10.1)
Black or African American	36 (33.0)	69 (30.4)	105 (31.3)
Hispanic or Latino	27 (24.8)	42 (18.5)	69 (20.5)
Native Hawaiian/Other Pacific Islander	—	1 (0.4)	1 (0.3)
White	15 (13.8)	23 (10.1)	38 (11.3)
Two or more races	9 (8.3)	17 (7.5)	26 (7.7)
Prefer to self-describe	1 (0.9)	1 (0.4)	2 (0.6)
Prefer not to say	2 (1.8)	5 (2.2)	7 (2.1)
Missing	5 (4.6)	46 (20.3)	51 (15.2)
Gender (*n* = 336)			
Woman	72 (66.1)	139 (61.2)	211 (62.8)
Man	33 (30.3)	46 (20.3)	79 (23.5)
Non-binary/gender non-conforming	—	2 (0.9)	2 (0.6)
Missing	4 (3.7)	40 (17.6)	44 (13.1)
Age range (*n* = 336)			
18–24	25 (22.9)	57 (25.1)	82 (24.4)
25–44	41 (37.6)	63 (27.8)	104 (31.0)
45–64	29 (26.6)	48 (21.1)	77 (22.9)
65+	9 (8.3)	9 (4.0)	18 (5.4)
Missing	5 (4.6)	50 (22.0)	55 (16.4)

**Table 2:** Associations between ArtsRx engagement level and program year, referral source, and participant characteristics.

ArtsRx program metrics	ArtsRx engagement level				
	No engagement % (*n*)	Low engagement % (*n*)	Medium engagement % (*n*)	High engagement % (*n*)	*p*-value
Completion status (*n* = 336)					
Completed	7.3 (8)	42.2 (46)	22.9 (25)	27.5 (30)	**< 0.001**
Withdrawn	90.3 (205)	7.5 (17)	2.2 (5)	0 (0)	
Program year (*n* = 336)					
Year 1	46.0 (58)	30.2 (38)	15.9 (20)	7.9 (10)	**< 0.001**
Year 2	73.8 (155)	11.9 (25)	4.8 (10)	9.5 (20)	
Referral source (*n* = 336)					
Horizon blue cross blue shield of NJ	56.0 (42)	26.7 (20)	12.0 (9)	5.3 (4)	**< 0.001** ^**#**^
Rutgers University-Newark	69.1 (114)	16.4 (27)	8.5 (14)	6.1 (10)	
RWJBarnabas health	25.0 (2)	37.5 (3)	37.5 (3)	0 (0)	
Returning citizens support group	59.3 (32)	11.1 (6)	1.9 (1)	27.8 (15)	
I am an NJPAC employee	41.7 (5)	33.3 (4)	16.7 (2)	8.3 (1)	
City of Newark department of health	81.8 (18)	13.6 (3)	4.5 (1)	0 (0)	
Race/ethnicity (*n* = 285)					
White	57.9 (22)	21.1 (8)	18.4 (7)	2.6 (1)	0.163
Black or African American	61.9 (65)	21.9 (23)	8.6 (9)	7.6 (8)	
Hispanic or Latino	52.2 (36)	24.6 (17)	13.0 (9)	10.1 (7)	
All Other races/ethnicities	63.0 (46)	13.7 (10)	6.8 (5)	16.4 (12)	
Gender (*n* = 290)					
Woman	59.7 (126)	22.7 (48)	10.0 (21)	7.6 (16)	0.098
Man	57.0 (45)	15.2 (12)	11.4 (9)	16.5 (13)	
Age range (*n* = 281)					
18–24	68.3 (56)	19.5 (16)	7.3 (6)	4.9 (4)	**0.052**
25–44	57.7 (60)	19.2 (20)	14.4 (15)	8.7 (9)	
45–64	51.9 (40)	23.4 (18)	10.4 (8)	14.3 (11)	
65+	44.4 (8)	27.8 (5)	0 (0)	27.8 (5)	

^**#**^Chi-square test performed despite low expected cell counts; Cramér's V and phi were also examined as measures of association.

#### Referral source and program year

Referral source, recorded at intake, was categorized as Horizon Blue Cross Blue Shield of New Jersey, Rutgers University–Newark, RWJBarnabas Health, Returning Citizens Support Group, NJPAC employees, or City of Newark Department of Health. Program year was defined as Year 1 (September 2023–August 2024) or Year 2 (September 2024–March 2025), corresponding to distinct implementation phases of ArtsRx.

#### Sociodemographic characteristics

Demographic characteristics were self-reported at intake and included age, race/ethnicity, and gender identity. Age was grouped as 18–24, 25–44, 45–64, and ≥65 years for descriptive reporting and retained in this form for bivariate analyses (Tables 2, 3). Race/ethnicity was initially collected using multiple categories. For cross-tabulations, values were recoded into four groups to address sparse cells: White; Black or African American; Hispanic or Latino; and All Other Races/Ethnicities (Asian, Arab/Middle Eastern/North African, Native Hawaiian or Other Pacific Islander, two or more races, and self-described categories). Participants with missing race/ethnicity were excluded from race/ethnicity-specific analyses. Gender identity response options included woman, man, non-binary/gender non-conforming, and prefer not to say. For bivariate analyses, gender was dichotomized as woman or man; participants identifying as non-binary/gender non-conforming or with missing gender were excluded from gender-specific tables due to the small cell size.

**Table 3:** Associations between referral source and program year and participant characteristics among ArtsRx participants.

ArtsRx program metrics	Referral source						
	Horizon blue cross blue shield of NJ % (*n*)	Rutgers University-Newark %(*n*)	RWJBarnabas health %(*n*)	Returning citizens support group %(*n*)	I am an NJPAC employee %(*n*)	City of Newark department of health %(*n*)	*p-value*
Completion status (*n* = 336)							0.164
Completed	23.9 (26)	43.1 (47)	1.8 (2)	20.2 (22)	6.4 (7)	4.6 (5)	
Withdrawn	21.6 (49)	52.0 (118)	2.6 (6)	14.1 (32)	2.2 (5)	7.5 (17)	
Program year (*n* = 336)							**< 0.001**
Year 1	40.5 (51)	50.0 (63)	5.6 (7)	4.0 (5)	0 (0)	0 (0)	
Year 2	11.4 (24)	48.6 (102)	0.5 (1)	23.3 (49)	5.7 (12)	10.5 (22)	
Race/ethnicity (*n* = 285)							**< 0.001** ^**#**^
White	34.2 (13)	44.7 (17)	2.6 (1)	2.6 (1)	10.5 (4)	5.3 (2)	
Black or African American	19.0 (20)	30.5 (32)	1.9 (2)	31.4 (33)	3.8 (4)	13.3 (14)	
Hispanic or Latino	30.4 (21)	50.7 (35)	5.8 (4)	8.7 (6)	1.4 (1)	2.9 (2)	
All Other races/ethnicities	17.8 (13)	64.4 (47)	1.4 (1)	9.6 (7)	2.7 (2)	4.1 (3)	
Gender (*n* = 290)							**< 0.001**
Woman	25.1 (53)	49.3 (104)	3.8 (8)	7.6 (16)	4.7 (10)	9.5 (20)	
Man	19.0 (15)	36.7 (29)	0 (0)	40.5 (32)	2.5 (2)	1.3 (1)	
Age range (*n* = 281)							**< 0.001** ^**#**^
18–24	4.9 (4)	89.0 (73)	0 (0)	1.2 (1)	2.4 (2)	2.4 (2)	
25–44	33.7 (35)	37.5 (39)	4.8 (5)	7.7 (8)	6.7 (7)	9.6 (10)	
45–64	32.5 (25)	20.8 (16)	3.9 (3)	31.2 (24)	2.6 (2)	9.1 (7)	
65+	5.6 (1)	11.1 (2)	0 (0)	77.8 (14)	5.6 (1)	0 (0)	

^**#**^Chi-square test performed despite low expected cell counts; Cramér's V and phi were also examined as measures of association.

### Data analysis

De-identified data were analyzed using SPSS (version 31). Descriptive statistics summarized program participation, engagement, referral source, and demographics. Bivariate associations between engagement level and program year, referral source, and participant characteristics were examined with chi-square tests of independence. Chi-square tests were also used to assess associations between referral source and program year and participant characteristics. When expected cell counts were low, chi-square results were interpreted cautiously, and effect size measures (Cramér's V and phi) were reviewed to gauge association strength. Statistical significance was defined as *p* < 0.05. Analyses used complete-case approaches for each comparison, yielding variable denominators across tables.

### Ethics

This study was reviewed and approved by the Rutgers University Institutional Review Board as exempt research due to the use of de-identified secondary data collected and organized by NJPAC. No individual consent was required.

## Results

A total of 498 individuals engaged with the ArtsRx program during the study period (Table 1). Of these, 21.9% (*n* = 109) completed the program, 45.7% (*n* = 227) withdrew before completion, 19.3% (*n* = 96) remained enrolled at the time of analysis, and 13.1% (*n* = 65) submitted an intake form but did not enroll. Analyses of engagement and participant characteristics focused on the 336 participants who either completed or withdrew from the program.

Within this analytic sample, Rutgers University–Newark was the predominant referral source, accounting for nearly half of participants (49.1%, *n* = 165), followed by Horizon Blue Cross Blue Shield of New Jersey (22.3%, *n* = 75). The remaining participants were referred through community-based organizations, health systems, and municipal partners, including the Returning Citizens Support Group, NJPAC, and the City of Newark Department of Health. Most participants enrolled during Year 2 of the program (62.5%, *n* = 210), with the remainder enrolling during Year 1 (37.5%, *n* = 126).

Engagement differed by completion status. Participants who completed the program attended a mean of 78.2% (SD = 30.1) of enrolled events and used 49.1% (SD = 33.9) of their allotted activities. In contrast, withdrawn participants attended 8.7% (SD = 27.3) of enrolled events and used 2.9% (SD = 10.1) of allotted activities. Nearly all withdrawn participants (90.3%) had no recorded event attendance, whereas completers were distributed across low (42.2%), medium (22.9%), and high (27.5%) engagement levels.

Sociodemographic characteristics were similar for participants who completed and those who withdrew (Table 1). Across the analytic cohort, participants most commonly identified as Black or African American (31.3%) or Hispanic or Latino (20.5%), and the majority identified as women (62.8%). Age spanned young to older adulthood, with approximately one third aged 25–44 years (31.0%), about one quarter aged 18–24 years (24.4%) and 45–64 years (22.9%), and a smaller proportion aged 65 years or older (5.4%). Missing demographic data were more frequent among withdrawn participants than among completers.

Table 2 highlights variation in ArtsRx engagement levels by program and participant characteristics. Program completion status was significantly associated with engagement level, with completers substantially overrepresented in the low, medium, and high engagement categories and withdrawn participants concentrated in the no-engagement group. This pattern reinforces the close link between sustained program engagement and eventual completion. Engagement level was also strongly associated with program year (*p* < 0.001). Among Year 1 enrollees, a smaller share had no engagement, and a larger share fell into low, medium, or high engagement categories, whereas Year 2 enrollees were more likely to have no engagement and less likely to achieve higher engagement levels. Engagement also varied by referral source (*p* < 0.001). Participants referred by the Returning Citizens Support Group and Rutgers University–Newark were more often represented in medium and high engagement categories than those referred by Horizon Blue Cross Blue Shield of New Jersey, health systems, or municipal agencies.

In contrast, engagement level did not differ meaningfully by race/ethnicity or gender. The distribution of engagement across racial and ethnic groups was similar (*p* = 0.163), and women and men showed comparable patterns of no, low, medium, and high engagement (*p* = 0.098). Age showed a borderline association with engagement (*p* = 0.052), with younger participants somewhat more likely to have no engagement and older participants somewhat more represented in higher engagement categories.

Table 3 describes how referral patterns varied across program year and participant characteristics. Completion status varied across referral sources but was not significant, with higher completion among participants referred by Rutgers University–Newark and the Returning Citizens Support Group and lower completion among those referred by municipal and health system partners. Referral source differed by program year, with a larger proportion of community-based and municipal referrals (e.g., Returning Citizens Support Group, City of Newark Department of Health) in Year 2 compared with Year 1 (*p* < 0.001). Referral source was also associated with race/ethnicity and age (both *p* < 0.001), with younger participants, particularly those aged 18–24 years, most often referred by Rutgers University–Newark, and older participants more frequently referred by community and municipal partners. Gender was related to referral patterns as well (*p* < 0.001), with women more often referred by Rutgers University–Newark and Horizon Blue Cross Blue Shield of New Jersey, and men more commonly referred through the Returning Citizens Support Group.

## Discussion

This study provides one of the first quantitative examinations of engagement patterns within a U.S.-based arts prescribing program in which program staff leverage a CRM platform for real-time tracking and longitudinal analysis. Findings from the first 2 years of ArtsRx implementation demonstrate both the promise and the challenges of operationalizing arts prescribing at scale within a complex, community-based ecosystem. While overall program completion rates were modest and withdrawal was common, participants who did complete the program exhibited substantially higher attendance and utilization of allotted activities, underscoring the importance of early and sustained engagement as a critical determinant of program success.

The scale of the ArtsRx program compared to other U.S. based pilots is worth highlighting here. The EpiArts Lab report documents that the majority of the 23 case study programs served between 0 and 50 participants, with several reporting extremely low uptake at the time of data collection: Zoo New England (Boston, MA) distributed 100 memberships but only 18 families redeemed them; Community Access to the Arts (Barrington, MA) reported a single monthly participant despite targeting 10 referrals per month; and the Norman Rockwell Museum (Stockbridge, MA) similarly reported one participating household per month. ArtsRx's reach of 498 individuals across 2 years, and its monthly referral volume across six distinct partner pathways, places it among the more developed ongoing programs in the U.S. landscape by scale (12).

The stark contrast in engagement between completers and withdrawn participants in ArtsRx mirrors patterns observed in broader social prescribing and community-based intervention literature, where attrition is often concentrated early in the intervention period (15, 16). While no U.S. arts prescribing program has published equivalent completion metrics, the pattern of low uptake and early disengagement is consistent across the EpiArts report and broader social prescribing literature (12). In the U.K., evaluations of social prescribing programs have documented that converting referrals to sustained participation is the primary challenge, with attrition heavily concentrated in the period between referral and first attendance (13, 17). Jensen et al.'s systematic review with meta-analysis of arts on prescription programs identifies low baseline wellbeing, multimorbidity, and structural barriers such as transportation and childcare as predictors of non-engagement (10). In ArtsRx, nearly all withdrawn participants had no recorded attendance, suggesting barriers to initial engagement rather than disengagement after participation begins. This finding has important implications for program design and quality improvement. It suggests that interventions targeting the “onboarding” phase, such as enhanced warm handoffs, more intensive follow-up immediately after referral, or peer navigation supports, may yield disproportionate gains in retention and completion. A similar emphasis on relational support during the early stages of engagement appears in evaluations of social prescribing programs, which highlight the central role of link workers and ongoing contact in converting referrals into sustained participation. Qualitative studies of link worker models suggest that trust building, individualized support, and practical problem solving, such as addressing transportation or scheduling barriers, can reduce early drop out and enhance participants' perceived value of social prescribing activities (13, 17).

Engagement varied significantly by program year, with Year 2 participants more likely to exhibit no engagement and less likely to achieve medium or high engagement levels compared with Year 1 participants. This pattern may reflect the realities of scaling-up the program. As ArtsRx expanded its referral base and diversified partnerships in Year 2, the program reached a broader and more heterogeneous population, including individuals likely facing more complex social and structural barriers to participation. Implementation science frameworks suggest that such “scale effects” are common, particularly when programs move from pilot phases to broader dissemination without commensurate increases in staffing, resources, or infrastructure (18, 19). Importantly, the use of a CRM platform enabled these shifts to be detected in near real time, highlighting the value of digital tools not only for evaluation but also for adaptive program management.

Referral source emerged as another modifier of engagement. Participants referred through community-embedded partners, such as Rutgers University–Newark and the Returning Citizens Support Group, more frequently demonstrated medium or high engagement compared with those referred by health system or insurer-based partners. These differences suggest that referral context may shape not only initial enrollment but also the likelihood of completing the ArtsRx program. This finding also aligns with prior research emphasizing the role of trust, relational continuity, and contextual alignment in social prescribing (8, 17, 20). Referrers who are embedded in participants' daily environments, such as educational institutions or community organizations serving specific populations, may be better positioned to frame arts prescribing as relevant, accessible, and personally meaningful. Specifically, this maps onto the EpiArts report's observation that “sites with limited or insecure funding expressed less capacity to build the kind of relationships necessary to broaden the reach of their program” and that relationship-building with referrers was a critical enabler across programs (12). Conversely, referrals originating from more institutional or bureaucratic contexts may require additional support to translate the referral into action. These results suggest that strengthening referrer training, clarifying expectations, and tailoring engagement strategies by referral pathway may enhance overall program effectiveness.

ArtsRx's six-pathway referral source model, spanning a health insurer, federally qualified health centers, a municipal health department, a university, and a community reintegration group is distinctive nationally. According to the literature, referral processes across U.S. programs are “almost always initiated by a healthcare professional, especially healthcare providers and mental health professionals,” and that most programs relied on one or two primary referral channels (12). This facilitated our ability to measure whether the type of referral source was a modifier of engagement, and, comparing against other social and arts prescribing literature, begin to make inferences on why.

Notably, engagement did not differ significantly by race/ethnicity or gender, and only borderline differences were observed by age. In a field where concerns about inequitable access and differential benefits are prominent, these findings are cautiously encouraging. They suggest that, once referred and enrolled, participants across demographic groups engaged with ArtsRx at broadly similar levels. At the same time, social prescribing evaluations have documented disparities in awareness, referral pathways, and capacity to take up offers, indicating that equity must be examined across the full referral-to-completion continuum, not just among enrolled participants (21, 22). Our analyses could not fully capture intersectional patterns or structural barriers such as transportation, caregiving responsibilities, or time constraints, underscoring the need for future mixed-methods work and ongoing equity monitoring using CRM data.

From a methodological standpoint, this study contributes to the growing literature on digital and data-enabled approaches to arts and social prescribing. Much of the existing evidence base relies on self-reported participation or retrospective recall, limiting precision and comparability across programs. The EpiArts report explicitly identifies technology for tracking referrals and monitoring engagement as a key enabler that was not universally present: several programs reported no formal evaluation process at the time of data collection, while others relied on end-of-year compiled surveys. Only Art Pharmacy describes a comparably systematic data infrastructure, using proprietary software for closed-loop reporting to referrers (12). By contrast, the ArtsRx CRM infrastructure allowed for standardized definitions of attendance and engagement, real-time data capture, and linkage across referral, enrollment, and participation stages. Because attendance and registration events are logged by NJPAC staff and partner organizations at the point of service rather than reported by participants after the fact, the CRM functions as an independent administrative record of engagement, reducing recall and social desirability bias that can affect self-reported measures. This approach supports more rigorous descriptive and inferential analyses and creates opportunities for future mixed-methods and longitudinal research examining how engagement trajectories relate to health, wellbeing, and healthcare utilization outcomes.

Overall, this analysis illustrates how a technology-enabled engagement tracking system for arts prescribing can generate actionable insights into participation patterns, identify leverage points for improving engagement, and support equity-focused implementation. As arts prescribing continues to expand across the U.S., investments in shared data infrastructure, common metrics, and cross-sector learning will be essential. Programs like ArtsRx, situated at the intersection of healthcare, community organizations, and cultural institutions, are well positioned to advance both the evidence base and the practical integration of the arts as a social driver of health.

### Limitations

Several limitations should be considered when interpreting these findings. First, analyses relied on observational data collected for program administration rather than research purposes, which may introduce misclassification, incomplete records, and variability in data entry across referral partners. Second, our analyses rely on administrative and participant-generated data on referrals, enrollment, and attendance, rather than direct reports of participants' experiences or perceived benefits. As a result, our engagement metrics capture whether and how often individuals attended ArtsRx activities, but they do not indicate whether those activities were experienced as enjoyable, meaningful, or simply convenient. Future work will incorporate qualitative interviews and survey-based feedback to better understand how participants interpret and experience arts engagement within ArtsRx. Third, the use of complete-case analyses resulted in varying analytic samples across comparisons, and small cell sizes for some subgroups limited the ability to examine intersectional patterns in engagement and referral pathways. Finally, ArtsRx is embedded within a specific regional and organizational context, which may limit the generalizability of these findings to social prescribing programs operating in different health systems or cultural settings.

## Conclusions

This study demonstrates the feasibility and analytic value of embedding a technology-enabled engagement tracking system within a community-based arts prescribing program in the U.S. Using 2 years of ArtsRx administrative data, we show that a CRM-based infrastructure can generate detailed, actionable insights into participation, engagement, and attrition across diverse referral pathways and participant populations. While overall completion rates were modest, participants who engaged meaningfully exhibited high attendance and utilization of available activities, underscoring the importance of early engagement as a key leverage point for program success. The observed absence of major demographic differences in engagement among enrolled participants is cautiously encouraging from an equity perspective, though these analyses cannot rule out unmeasured inequities in access or retention.

Beyond the specific findings of ArtsRx, this study contributes to the broader arts and health and social prescribing literature by illustrating how digital tools can strengthen implementation science in this field. Standardized, real-time engagement metrics enable programs to move beyond anecdotal evidence and self-reporting, support continuous quality improvement, and lay the groundwork for more rigorous evaluations of health, wellbeing, and cost-related outcomes. In addition to supporting research, these data can inform internal quality improvement by highlighting stages in the participation process where disengagement is most common, such as between referral, enrollment, and first attendance. Identifying these points can help programs implement targeted changes to onboarding, warm handoffs, and follow-up procedures to improve retention and completion. As similar arts prescribing initiatives adopt shared engagement metrics and CRM-based monitoring, programs may be better equipped to refine referral pathways, encourage fuller participation in available activities, and ultimately increase completion rates. The integration of shared data infrastructure and common engagement measures will be critical for comparability, scalability, and policy relevance, as arts prescribing initiatives continue to proliferate across the U.S.

Future research should build on this foundation by incorporating qualitative methods to better understand barriers and facilitators to initial engagement, examining longitudinal re-enrollment and sustained participation, and linking engagement trajectories to downstream health and social outcomes. From a practice and policy standpoint, investments in referral relationship-building, participant onboarding, and data-enabled program management may yield substantial returns. Collectively, these findings position technology-enabled arts prescribing models like ArtsRx as a promising, equity-oriented approach to integrating the arts as a social driver of health within evolving health and community care systems.

## Data Availability

To protect the confidentiality of our participants, we do not make data freely available. Data are housed at the Rutgers School of Public Health's Center for Health, Identity, Behavior and Prevention Studies (CHIBPS), and the data set may be made available upon written request to the corresponding author.
