# Supplementary material for: ArtsRx as an arts prescribing model: leveraging CRM data to understand participation and engagement
Source: Front Public Health. 2026 Jun 10;14:1800466. doi: 10.3389/fpubh.2026.1800466 (PMC13290757; doi:10.3389/fpubh.2026.1800466)
Supplement: Supplementary file 1 [file Table_1.doc]

**Supplementary Table 1. STROBE Statement completed checklist**

|  | Item No | Recommendation | Our Paper |
| --- | --- | --- | --- |
| **Title and abstract** | 1 | (*a*) Indicate the study’s design with a commonly used term in the title or the abstract | "Retrospective observational study" in Methods sentence of Abstract; Methods first paragraph. |
|  |  | (*b*) Provide in the abstract an informative and balanced summary of what was done and what was found | Abstract summarizes background, aims, design (retrospective observational), data sources, main results (completion 21.9%, engagement modifiers), conclusions. |
| Introduction | | |  |
| Background/rationale | 2 | Explain the scientific background and rationale for the investigation being reported | Introduction paragraphs 1–4: social/arts prescribing context, engagement tracking gaps, ArtsRx + CRM rationale. ​ |
| Objectives | 3 | State specific objectives, including any prespecified hypotheses | Final Introduction paragraph: three aims (describe patterns; assess variation by year/referral/demographics; CRM implications). |
| Methods | | |  |
| Study design | 4 | Present key elements of study design early in the paper | "Study Design and Participants" subsection: retrospective observational study of de-identified administrative/survey data. |
| Setting | 5 | Describe the setting, locations, and relevant dates, including periods of recruitment, exposure, follow-up, and data collection | "Study Design and Participants" and "ArtsRx Referral Pathway": Newark/Essex County, NJ, U.S.; referral partners; September 2023–March 2025. |
| Participants | 6 | (*a*) Give the eligibility criteria, and the sources and methods of selection of participants | "Study Design and Participants": all 498 referrals recorded in CRM; analytic n=336 (completed/withdrawn) for engagement; no formal eligibility beyond referral. ​ |
| Variables | 7 | Clearly define all outcomes, exposures, predictors, potential confounders, and effect modifiers. Give diagnostic criteria, if applicable | "Measures" subsections: defines engagement/attendance rates, categories (no/low/medium/high), referral source, program year, age/race/gender groupings. ​ |
| Data sources/ measurement | 8* | For each variable of interest, give sources of data and details of methods of assessment (measurement). Describe comparability of assessment methods if there is more than one group | "Study Design and Participants": Salesforce CRM (real-time attendance/enrollment), Formstack/Qualtrics surveys (demographics); staff-recorded data. |
| Bias | 9 | Describe any efforts to address potential sources of bias | "Limitations": discusses administrative data quality (misclassification, incomplete records); no formal bias mitigation; acknowledges limitations. |
| Study size | 10 | Explain how the study size was arrived at | "Study Design and Participants": includes all 498 referrals in dataset; analytic n=336 for engagement. |
| Quantitative variables | 11 | Explain how quantitative variables were handled in the analyses. If applicable, describe which groupings were chosen and why | "Measures" and "Data Analysis": engagement rate as proportion; categorized into 4 levels; age into 4 groups; race recoded to 4 groups; gender dichotomized. |
| Statistical methods | 12 | (*a*) Describe all statistical methods, including those used to control for confounding | "Data Analysis": chi-square tests; p<0.05; no confounding adjustment (descriptive/bivariate). ​ |
|  |  | (*b*) Describe any methods used to examine subgroups and interactions | "Data Analysis" and Tables 2–3: chi-square cross-tabulations by engagement level across year, referral, demographics. ​ |
|  |  | (*c*) Explain how missing data were addressed | "Data Analysis" and "Limitations": complete-case analyses; varying denominators; higher missingness among withdrawn participants. |
|  |  | (*d*) If applicable, describe analytical methods taking account of sampling strategy | N/A |
|  |  | (*e*) Describe any sensitivity analyses | N/A |
| Results | | |  |
| Participants | 13* | (a) Report numbers of individuals at each stage of study—eg numbers potentially eligible, examined for eligibility, confirmed eligible, included in the study, completing follow-up, and analysed | "Results" and Table 1: 498 total; % completed (21.9%), withdrew (45.7%), enrolled (19.3%), intake only (13.1%); analytic n=336. |
|  |  | (b) Give reasons for non-participation at each stage | "Results" and Table 1: withdrew (unresponsive >2 months); intake only (no enrolment); higher missing demographics among withdrawn. |
|  |  | (c) Consider use of a flow diagram | Table 1 serves as flow summary. |
| Descriptive data | 14* | (a) Give characteristics of study participants (eg demographic, clinical, social) and information on exposures and potential confounders | Table 1: demographics, referral sources, participation status; Results text summarizes. |
|  |  | (b) Indicate number of participants with missing data for each variable of interest | Table 1 and "Results": missing demographics higher among withdrawn (e.g., 22% missing age); excluded from specific analyses. ​ |
| Outcome data | 15* | Report numbers of outcome events or summary measures | Tables 1–3: attendance/engagement rates by completion status; engagement categories by year, referral, demographics. ​ |
| Main results | 16 | (*a*) Give unadjusted estimates and, if applicable, confounder-adjusted estimates and their precision (eg, 95% confidence interval). Make clear which confounders were adjusted for and why they were included | Tables 2–3: chi-square p-values and % distributions for engagement associations; no adjusted estimates (descriptive). |
|  |  | (*b*) Report category boundaries when continuous variables were categorized | "Measures": engagement levels (0=no, 1–2=low, 3–4=medium, 5+=high); age groups (18–24, 25–44, etc.). |
|  |  | (*c*) If relevant, consider translating estimates of relative risk into absolute risk for a meaningful time period | N/A |
| Other analyses | 17 | Report other analyses done—eg analyses of subgroups and interactions, and sensitivity analyses | Discussion interprets program-year/referral patterns and equity findings; no additional formal analyses. |
| Discussion | | |  |
| Key results | 18 | Summarise key results with reference to study objectives | Discussion first paragraph: summarizes completion rates, engagement modifiers, early attrition, onboarding implications. |
| Limitations | 19 | Discuss limitations of the study, taking into account sources of potential bias or imprecision. Discuss both direction and magnitude of any potential bias | "Limitations" section: administrative data quality, missing data, no subjective outcomes, small subgroups, regional context. |
| Interpretation | 20 | Give a cautious overall interpretation of results considering objectives, limitations, multiplicity of analyses, results from similar studies, and other relevant evidence | Discussion paragraphs 2–6: contextualizes findings against social prescribing literature, equity, implementation science. |
| Generalisability | 21 | Discuss the generalisability (external validity) of the study results | "Limitations" and Conclusions: notes specific Newark/NJPAC context may limit generalisability to other settings. |
| Other information | | |  |
| Funding | 22 | Give the source of funding and the role of the funders for the present study and, if applicable, for the original study on which the present article is based | "Methods" (Methods and Materials, near end): Rutgers OVPR Behavioral Health and Equity Pilot Seed Funding; NJPAC institutional/philanthropic support for program; funders had no role in analysis. |
